# Development of a Computational Histology Artificial Intelligence-Powered Prognostic Biomarker in Colorectal Cancer in The Cancer Genome Atlas

**DOI:** 10.14740/wjon2823

**Published:** 2026-09-04

**Authors:** Chris Lieu, Vivek Nimgaonkar, Viswesh Krishna, Trevor J. Royce, Richard M. Goldberg

**Affiliations:** a Department of Medical Oncology, University of Colorado, Aurora, CO, USA; b Department of Medical Oncology, Johns Hopkins University, Baltimore, MD, USA; c Valar Labs, Palo Alto, CA, USA; d Department of Radiation Oncology, University of North Carolina at Chapel Hill School of Medicine, Chapel Hill, NC, USA; e West Virginia University Cancer Institute, Morgantown, WV, USA

**Keywords:** Colorectal cancer, Artificial intelligence, Computational pathology, Biomarker, Prognostic, TCGA, Histopathology

## Abstract

**Background:**

Risk stratification in colorectal cancer (CRC) plays an important role in treatment decision-making. As such, prognostic biomarkers that can augment risk stratification have clinical value. Quantitative histologic features from routine hematoxylin and eosin (H&E)-stained whole slide images (WSIs) provide a novel avenue for biomarker discovery. In this study, we explored the potential for a computational histology artificial intelligence (CHAI) platform to develop and validate a prognostic biomarker in CRC.

**Methods:**

The Cancer Genome Atlas Colorectal Adenocarcinoma project was utilized for this study, with inclusion of all subjects (stage I–IV) with available digitized H&E specimens. The cohort was split into development and validation cohorts by a stratified random split. The previously developed CHAI platform was applied in the development cohort to construct a continuous risk score from histologic features associated with progression-free interval (PFI) that was dichotomized based on an optimized cutpoint for distinguishing PFI into a high risk CHAI (+) and lower risk CHAI (–). PFI was compared between CHAI (+) and CHAI (–) patients in the validation cohort in multivariable Cox proportional hazards models. Time-dependent area under the curve (tdAUC) and C-indices were also calculated for PFI.

**Results:**

A total of 583 participants were included in the study, with 409 assigned to the validation cohort. The CHAI biomarker classified 229 participants (56%) as CHAI (+) and 180 (44%) as CHAI (–) in the validation set. CHAI (+) participants had worse PFI in a multivariable analysis adjusting for available clinicopathologic variables (hazard ratio (HR) = 2.65; 95% confidence interval (CI), 1.63–4.30). TdAUC for the CHAI biomarker was 0.60 (95% CI, 0.53–0.67) at 12 months, 0.62 (0.55–0.69) at 36 months, and 0.67 (0.55–0.79) at 60 months; the C-index was 0.62 (95% CI, 0.58–0.67).

**Conclusions:**

The CHAI platform was used to develop a prognostic digital pathology biomarker in CRC. This demonstrates the feasibility and potential to apply this artificial intelligence-based digital pathology biomarker platform for risk stratification in CRC and supports its further study.

## Introduction

Colorectal cancer (CRC) accounts for more than 900,000 deaths annually across the world, with an increasing incidence of disease among younger age groups [1]. The majority of patients with CRC have localized disease at diagnosis, and in such cases, the mainstay of treatment is surgical resection with consideration for additional adjuvant systemic therapy that can reduce the risk of recurrence [2, 3]. Importantly, even in the metastatic setting, a subset of patients with limited burden of metastatic disease can undergo definitive resection with curative intent [4]. Across the disease spectrum, careful risk stratification is necessary to guide treatment selection, for instance in the determination of when to administer and in the selection of adjuvant therapy for stage II or III disease [5]. Increasingly, molecular testing and blood-based biomarkers (such as circulating tumor DNA) are being utilized to select treatments and augment tumor characterization [6]. These tools complement traditional pathologic characteristics that have historically identified higher risk tumors, such as differentiation status, lymphovascular invasion (LVI), perineural invasion, and tumor budding [7, 8].

Computational pathology approaches that involve the use of artificial intelligence methods in analysis of digitized histopathology images represent an emerging biomarker modality [9]. Such applications have been applied in CRC to identify biomarkers associated with prognosis [10–12]. One tumor-agnostic tool for digital pathology analysis is the computational histology artificial intelligence (CHAI) platform [13]. The CHAI platform has previously been applied in bladder cancer, pancreatic cancer, and prostate cancer to develop prognostic and treatment-predictive biomarkers from digitized hematoxylin and eosin (H&E) tissue specimens [13–17]. The platform is built upon a deep learning system of tissue and cell segmentation models that extract over 30,000 histomorphological features that can be compiled into biomarker signatures associated with outcomes of interest.

In this study, we sought to apply the CHAI platform in the setting of CRC to assess whether it could be used to identify a novel prognostic biomarker with data from The Cancer Genome Atlas (TCGA). Dividing the available TCGA colorectal adenocarcinoma projects into a development and validation cohort, we first developed a continuous risk score from a set of histologic features associated with worse outcomes with subsequent dichotomization of the score into low-risk and high-risk biomarker strata. We explored the associations of this binary biomarker with other clinicopathologic variables in a validation cohort and tested the performance of the biomarker in stratifying patients by progression-free interval (PFI).

## Materials and Methods

### Study cohort

This study utilized data from The Cancer Genome Atlas colorectal adenocarcinoma projects (TCGA-COAD and TCGA-READ), multi-institutional efforts that collected primary CRC specimens with corresponding clinical, genomic, and molecular data [18]. Participants were included if they had: (1) available digitized whole slide images (WSIs) of H&E-stained diagnostic specimens that passed quality control on the CHAI platform; and (2) available clinical survival data. All specimens were obtained from primary tumor resection. Institutional Review Board (IRB) review and approval were waived for this study as this was a retrospective analysis of publicly available deidentified data from TCGA without protected health information (PHI). The study was conducted in compliance with the ethical standards of the responsible institution on human subjects as well as with the Helsinki Declaration.

### Study endpoints

The primary endpoint was PFI, which has previously been defined as the time from initial diagnosis to disease progression, locoregional recurrence, distant metastasis, new primary tumor, or death [19]. Participants without a documented progression event were censored at the date of last known follow-up. Overall survival (OS), defined as the time from initial pathologic diagnosis to death from any cause, was evaluated as a secondary exploratory endpoint.

### Study design

Participants were divided based on a stratified random split by stage into a development set (30%) and a validation set (70%). A 30:70 split allowed for a development cohort size similar to prior work where the CHAI platform was implemented for biomarker development in another gastrointestinal cancer [16]. The CHAI biomarker classification signature was developed and optimized on the development set. Features and weights were then locked, and the biomarker was applied without modification to the validation set. All primary results are reported from the validation set.

The previously described CHAI platform was applied to digitized WSI from the TCGA CRC cohort. Briefly, the platform processes H&E-stained slides through deep learning models to segment tissue and cell regions, extracting quantitative histomorphological features related to phenotypic attributes of cancer biology, including nuclei size and morphology, cell spatial orientation, host immune cell infiltration, and stromal density. The CRC biomarker is derived exclusively from H&E histopathology images without clinical variable inputs. Quantitative features were selected for inclusion into a biomarker signature based on univariable Cox proportional hazards (CPH) analysis of individual features. Features included in the signature described tumor nucleus aggressiveness, tumor proliferation, and degree of immune infiltration. Feature weightings in the signature were determined based on univariable CPH results for individual features, and the signature was further refined using multivariable CPH models and chunk tests. The resulting output of this biomarker signature was a continuous risk score that was generated for each participant. A binary CHAI biomarker classification was derived from the continuous risk score: CHAI (+) indicating higher predicted risk and CHAI (–) indicating lower predicted risk. The median risk score value was used as the threshold for the biomarker classification threshold with the rationale that a median cutpoint would maximize statistical power with balanced CHAI (+) and CHAI (–) groups in the absence of an alternative biologic rationale for a particular cutpoint. The biomarker was locked based on the development cohort, and it was then applied to the held-out validation set without modification.

### Statistical analysis

Patient demographics and clinical characteristics were summarized using descriptive statistics. Medians and interquartile ranges (IQRs) were reported for continuous variables, and counts with proportions were reported for categorical variables. The association of the CHAI biomarker with PFI and OS was evaluated using CPH models. Univariable models assessed the unadjusted association of the CHAI biomarker with each endpoint. The primary multivariable CPH model included the CHAI biomarker, age at diagnosis, sex, American Joint Committee on Cancer (AJCC, seventh edition) pathologic stage, LVI, sidedness, and the number of nodes examined, as covariates. Microsatellite instability (MSI) status and perineural invasion status were available for only small subsets of participants and were therefore not included in primary multivariable models. Tumor grade was not available in the dataset. Hazard ratios (HR), 95% confidence intervals (CIs), and P values were reported. Time-dependent area under the curve (tdAUC) and the Harrell’s C-index for the univariable CPH biomarker model of PFI were also calculated to describe biomarker performance in the validation cohort. Kaplan–Meier estimators were used to report biomarker performance in stratifying outcomes in stage II and III disease where risk stratification has traditionally been of interest for decision-making around the use of adjuvant chemotherapy. To address data missingness, multiple imputation by chained equations was performed, generating 20 imputed datasets. A pre-specified significance threshold of P < 0.05 was used. All statistical analyses were performed with R (R Foundation, Vienna, Austria).

## Results

### Patient characteristics

A total of 583 participants from the TCGA CRC cohort met inclusion criteria and were included in this analysis. Of these, 174 (30%) were randomly assigned to the development set and 409 (70%) to the validation set. Clinical characteristics were balanced between the two sets (Table 1).

**Table 1:** Clinical Characteristics of Patients in the Development and Validation Cohorts

Characteristic	Overall (n = 583)^a^	Development (n = 174)^a^	Validation (n = 409)^a^	P value^b^
Age (years)	68.0 (58.0–76.0)	66.5 (59.0–75.0)	68.0 (57.0–76.0)	0.78
Sex				0.10
Female	278 (48%)	92 (53%)	186 (45%)	
Male	305 (52%)	82 (47%)	223 (55%)	
Race				0.60
White	275 (78%)	75 (77%)	200 (79%)	
Black	63 (18%)	21 (21%)	42 (17%)	
Asian	12 (3%)	2 (2%)	10 (4%)	
American Indian	1 (0%)	0 (0%)	1 (0%)	
Missing	232	76	156	
AJCC stage				1.00
I	107 (18%)	32 (18%)	75 (18%)	
II	213 (37%)	64 (37%)	149 (36%)	
III	180 (31%)	54 (31%)	126 (31%)	
IV	83 (14%)	24 (14%)	59 (14%)	
Microsatellite instability (MSI) status				0.73
MSS	103 (91%)	34 (89%)	69 (92%)	
MSI-H	10 (9%)	4 (11%)	6 (8%)	
Missing	470	136	334	
Sidedness				0.85
Left	318 (56%)	96 (56%)	222 (57%)	
Right	246 (44%)	76 (44%)	170 (43%)	
Missing	19	2	17	
Lymphovascular invasion				0.22
Absent	300 (55%)	96 (59%)	204 (53%)	
Present	244 (45%)	66 (41%)	178 (47%)	
Missing	39	12	27	
Perineural invasion				0.40
Absent	165 (74%)	50 (78%)	115 (72%)	
Present	58 (26%)	14 (22%)	44 (28%)	
Missing	360	110	250	
Lymph nodes examined	19.0 (14.0–27.0)	20.0 (15.0–28.0)	19.0 (14.0–27.0)	0.12
Missing	27	9	18	

^a^Median (Q1–Q3); n (%). ^b^Wilcoxon rank sum test; Fisher’s exact test. Patients were randomly partitioned into a development cohort (n = 174) and validation cohort (n = 409). Clinical characteristics of patients in both cohorts are summarized. Continuous variables are summarized as median (interquartile range) and categorical variables as n (%). Between cohort differences were tested using the Wilcoxon rank-sum test for continuous variables and Fisher’s exact test for categorical variables. AJCC: American Joint Committee on Cancer; MSI: microsatellite instability; MSS: microsatellite stable: MSI-H: microsatellite instability-high.

In the validation set (n = 409), the median age at diagnosis was 68 years (IQR 57–76). Of the participants, 223 (55%) were male, and 186 (45%) were female. Race data were available for 253 participants (62%); 200 (49%) were White, 42 (10%) were Black or African American, 10 (2%) were Asian, and one (< 1%) was American Indian or Alaska Native. The distribution of AJCC pathologic stages was: stage I in 75 (18%), stage II in 149 (36%), stage III in 126 (31%), and stage IV in 59 (14%). Among the tumors, 293 (72%) were colon primaries and 112 (27%) were rectal primaries. By sidedness, 170 (41%) were right-sided and 222 (54%) were left-sided. LVI was present in 178 (44%), absent in 204 (50%), and not reported in 27 (7%). MSI status was available for only 75 participants (18%); among these, six were MSI-high and 69 were microsatellite stable.

At a median follow-up of 22.0 months among censored patients, 111 PFS events (27%) and 89 deaths (22%) were observed in the validation set. In the development set, 40 PFS events (23%) and 32 deaths (18%) were observed at a median follow-up of 22.1 months.

### Association of the CHAI biomarker with clinicopathologic characteristics

**Figure 1:**
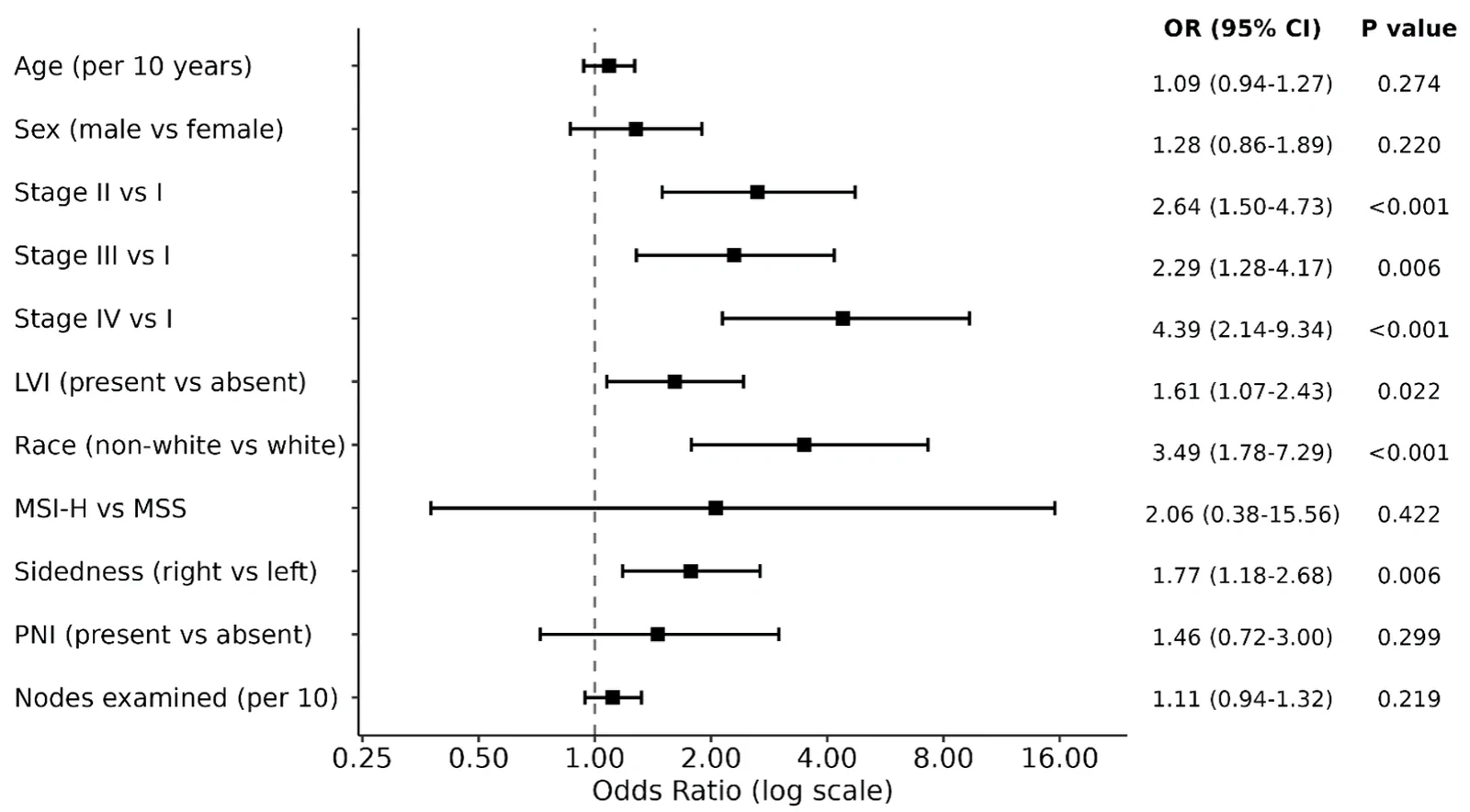
Associations between clinical variables and CHAI biomarker status. ORs (squares) and 95% confidence intervals (horizontal bars) were estimated from individual logistic regression models in the validation cohort (n = 409), with CHAI biomarker (+) status. ORs to the right of the vertical dashed line (OR = 1) indicate increased odds of biomarker positivity. Reference categories: female (sex), AJCC stage I (stage), absent (LVI, PNI), White (race), MSS (MSI status), left (sidedness). AJCC: American Joint Committee on Cancer; CHAI: computational histology artificial intelligence; CI: confidence interval; LVI: lymphovascular invasion; MSI-H: microsatellite instability-high; MSS: microsatellite stable; OR: odds ratio; PNI: perineural invasion.

The CHAI biomarker classified 229 participants (56%) as CHAI (+) and 180 (44%) as CHAI (–) in the validation set. Among available patient characteristics for assessment, stage, presence of LVI, race, and sidedness were associated with CHAI biomarker status (Fig. 1). Stage was correlated with CHAI biomarker status, with higher proportions of CHAI (+) patients in later stages of disease: stage I CHAI (+) in 27/48 (36%), stage II CHAI (+) in 89/149 (60%), stage III CHAI (+) in 71/126 (56%), stage IV CHAI (+) in 42/59 (71%). The presence of LVI was associated with higher odds of CHAI (+) status (odds ratio (OR) = 1.61; 95% CI, 1.07–2.42; P = 0.022). CHAI (+) status was more frequent among non-White patients (OR = 3.49; 95% CI, 1.73–7.02; P < 0.001). Right-sided CRCs were also more frequently CHAI (+) (OR = 1.77; 95% CI, 1.18–2.67; P = 0.006).

### Association of CHAI biomarker with PFI

**Figure 2:**
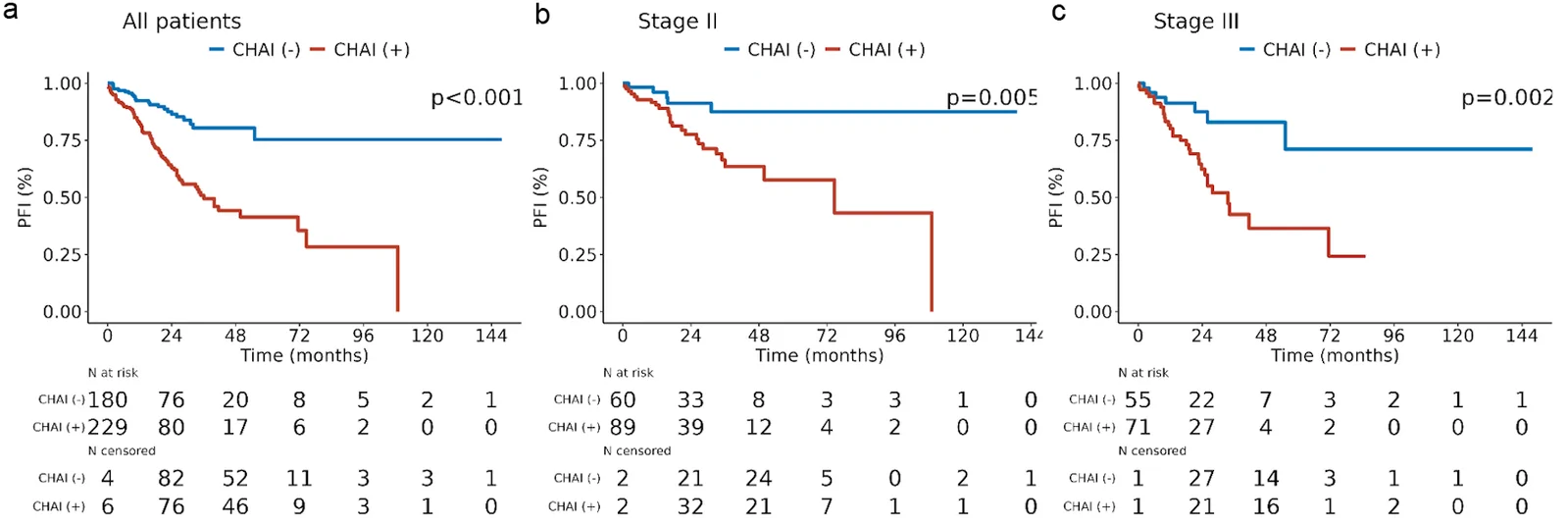
PFI by CHAI biomarker status. Kaplan–Meier estimates of PFI are stratified by CHAI status in (a) all validation cohort patients (n = 409; one patient in the validation cohort did not have available PFI data), (b) stage II patients (n = 150), and (c) stage III patients (n = 126). P values correspond to log-rank tests. AJCC: American Joint Committee on Cancer; CHAI: computational histology artificial intelligence; CI: confidence interval; PFI: progression-free interval.

**Figure 3:**
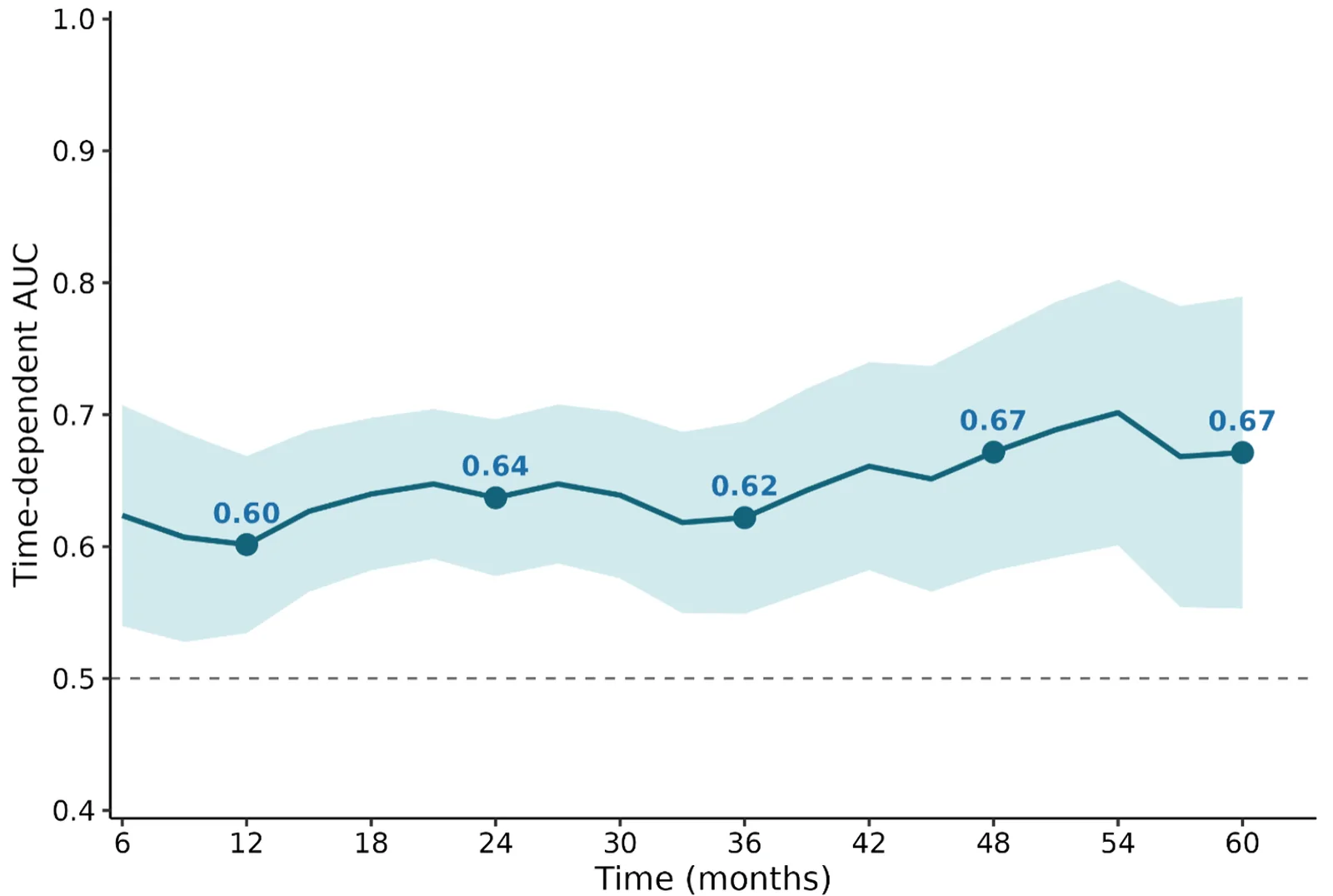
Time-dependent AUC for progression free interval of the CHAI biomarker. Time-dependent AUC for CHAI status in predicting PFI, evaluated at 3-month intervals from 6 to 60 months in the validation cohort (n = 409). The solid line shows the point estimate with the shaded band representing the 95% confidence interval. Filled circles mark the AUC at the 1-, 2-, 3-, 4-, and 5-year time points. AUC: area under the curve; CHAI: computational histology artificial intelligence; CI: confidence interval; PFI: progression-free interval.

On univariable analysis in the validation set, the CHAI biomarker was associated with PFI (HR = 3.41; 95% CI, 2.15–5.41; P < 0.001; median PFI: CHAI (+) not reached (95% CI: not reached–not reached) vs CHAI (–) 36.0 months (95% CI, 27.8 months–not reached)) (Fig. 2a). When looking specifically at stage II and III disease, where risk stratification frequently informs adjuvant therapy selection and duration, CHAI (+) status was consistently associated with worse PFI for stage II and III disease (stage II: HR = 3.65; 95% CI, 1.38–9.65; log-rank P = 0.005; stage III: HR =3.52; 95% CI, 1.54–8.04; log-rank P = 0.002) (Fig. 2b, c). As a benchmark pathologic variable with known association with worse prognosis, LVI was associated with worse PFI among all patients (log-rank test P < 0.001) (Supplementary Material 1a, wjon.elmerpub.com). However, LVI was not associated with worse PFI when specifically examining stage II and III disease (Supplementary Material 1b, c, wjon.elmerpub.com). The tdAUCs for the CHAI biomarker was 0.60 (95% CI, 0.53–0.67) at 12 months, 0.62 (95% CI, 0.55–0.69) at 36 months, and 0.67 (0.55–0.79) at 60 months (Fig. 3). The C-index for the univariable CPH of PFI with the CHAI biomarker was 0.62 (95% CI, 0.58–0.67).

### Multivariable analysis of the CHAI biomarker with PFI

**Figure 4:**
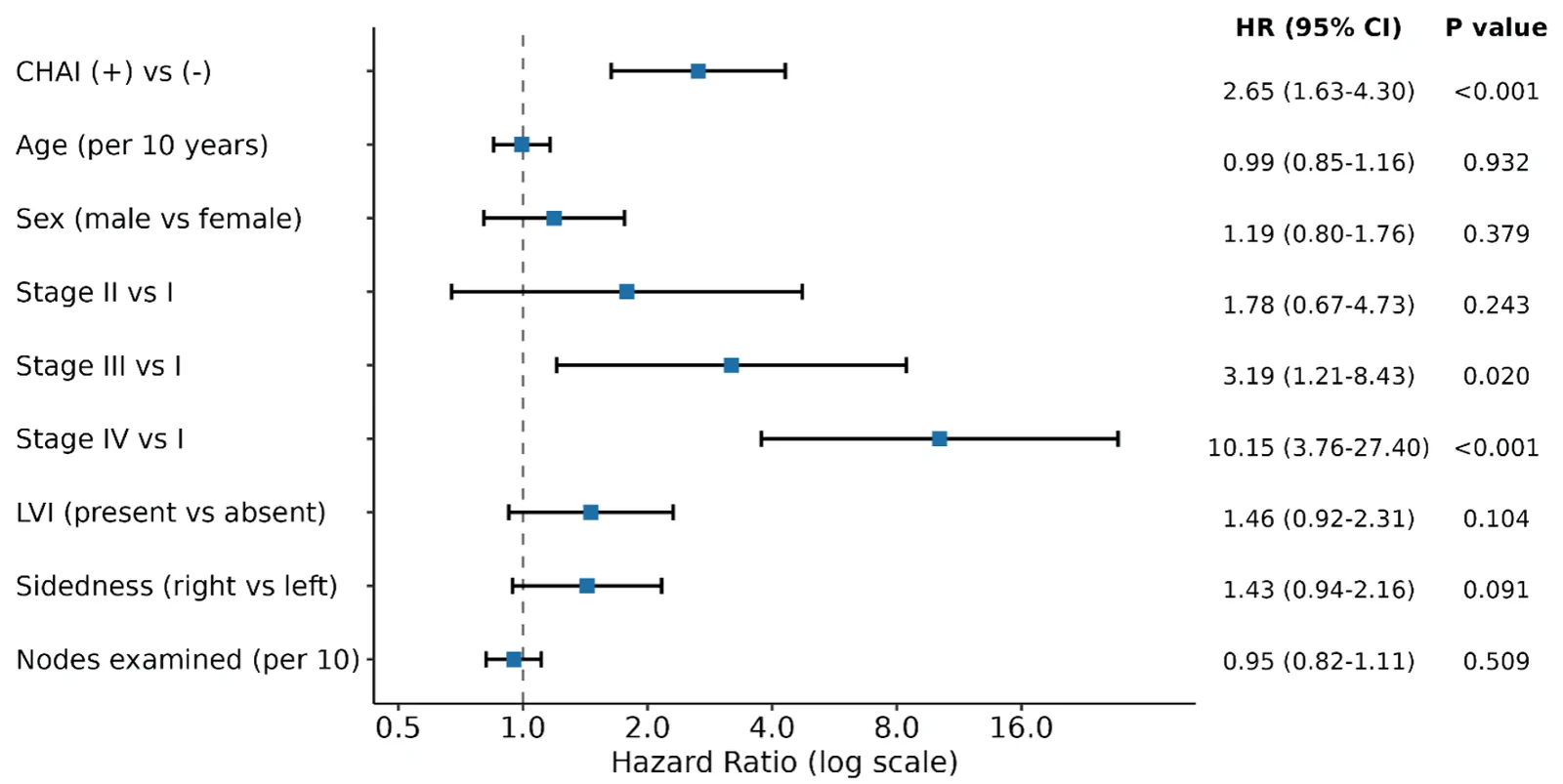
Multivariable cox proportional hazards model of PFI. Hazard ratios (squares) and 95% confidence intervals (horizontal bars) for the association of each covariate with PFI in a multivariable cox proportional hazards model in the validation cohort (n = 409 with PFI data). Missing values for covariates were handled by multiple imputation (20 imputations). Reference categories were: CHAI (–) (CHAI status), female (sex), stage I (stage), absent (lymphovascular invasion), left-sided (sidedness). aHR: adjusted hazard ratio; AJCC American Joint Committee on Cancer; CHAI: computational histology artificial intelligence; CI: confidence interval; LVI: lymphovascular invasion; PFI: progression-free interval.

A multivariable CPH model for PFI, including the CHAI biomarker, age, sex, stage, LVI, sidedness and nodes examined, was composed for participants in the validation cohort. CHAI (+) remained associated with worse PFI after adjustment in this model (HR = 2.65; 95% CI, 1.63–4.30; P < 0.001). Only stage III and stage IV disease were also significantly associated with PFI, aside from CHAI biomarker status (Fig. 4).

### Exploratory analysis of OS

Limited follow-up data were available to study OS, with only 89 OS events in the validation cohort. Nevertheless, we performed an exploratory analysis for an association between CHAI biomarker status and OS. In a univariable CPH model, CHAI (+) status was associated with worse OS (HR = 1.78; 95% CI, 1.14–2.81; P = 0.012). In a multivariable model including CHAI biomarker status, age, stage, LVI, sidedness, and nodes examined, the association of CHAI (+) status with OS was attenuated and no longer significant (HR = 1.24; 95% CI, 0.75–2.04; P = 0.403).

## Discussion

In this study, the CHAI platform was applied in the context of CRC. Using data from the TCGA, it was feasible to develop and validate an AI-powered digital pathology biomarker associated with worse PFI. The biomarker is associated with later stage disease, LVI, and right-sided primary site, but the prognostic association of CHAI biomarker status with PFI was found to be independent of these variables.

Risk stratification represents an important part of treatment decision-making in management of CRC. In this context, the association of the CHAI biomarker with prognosis is notable and suggests that the CHAI platform can be applied effectively to augment risk stratification. The HR of 2.65 for the CHAI (+) status suggests that CHAI (+) disease is associated with more than two-fold greater risk of progression. LVI by contrast was not significantly associated with worse PFI. The CHAI biomarker was found to be associated with several other known prognostic clinical characteristics, including stage, LVI, and right-sided disease; however, the independence of the CHAI biomarker from these characteristics in a multivariable suggests that it provides distinct prognostic information.

Decisions about adjuvant chemotherapy in early-stage disease are particularly reliant upon effective risk stratification. In stage II disease, assessment of high-risk features, including pathologic characteristics such as LVI, perineural invasion, and differentiation status, is used to inform whether to recommend adjuvant chemotherapy. In stage III disease, the IDEA study suggested that duration of adjuvant platinum chemotherapy could reasonably be shortened for certain lower risk tumors [20]. Given the clinical importance of risk stratification in stage II and III disease, it is striking that CHAI biomarker status stratifies patients by outcomes when looking at stage II and stage III patients in isolation. Circulating tumor DNA has increasingly been studied as a tool in stage II and stage III disease, with promise as a tool to stratify patients by prognosis [21–23]. Digital pathology as a distinct data modality may offer a complementary biomarker approach to circulating tumor DNA to optimize prognostication and to create an accurate composite risk model for prospective phase II/III studies. Further study is warranted in datasets with greater statistical power and more information about treatment, but the CHAI biomarker may offer one additional characteristic that marks high-risk disease, particularly in resectable disease where risk stratification sharpens decisions such as whether and for how long to administer adjuvant therapy.

The association of the CHAI biomarker with OS was not robust to multivariable analysis in this study. This could be related to the limited duration of the available follow-up data: median follow-up of 22.0 months likely is an insufficient interval for assessment of OS, particularly in earlier stage disease. Additionally, OS can be heavily influenced by access to and utilization of subsequent lines of therapy, and this dataset did not have granular information to address this. Further evaluation in cohorts with longer term follow-up and more advanced disease is needed to assess the OS association with the CHAI biomarker.

Digital pathology approaches have previously been utilized in CRC to enhance or automate the histopathologic diagnosis of CRC [24]. Prediction of MSI status with computational pathology methods has proven feasible, along with assessment of mutational status [25, 26]. An array of tools has been developed and validated with impressive performance characteristics for prognostic purposes, including DoMore-v1-CRC, AImmunoscore, DGMuneS, HIBRID (incorporating ctDNA), Pathomics Signature, and Tumor Adipose Feature, amongst others [27–32]. This study represents a further entry into this space, supporting the capacity for computational pathology approaches to enhance risk stratification. The novelty of this study rests in the application of a computational pathology platform developed in a different disease context.

This study has several limitations. First, this represented an initial study without external validation using the TCGA data with a convenience sample from this retrospective multi-institutional repository. The lack of long-term follow-up and limited scope to evaluate OS are additional limitations. While PFI has been recognized as a valid outcome measure in TCGA analyses, evaluation of the CHAI biomarker across other outcomes of interest would be desirable. Other pathologic characteristics such as perineural invasion, differentiation status, tumor budding, and MSI status were also largely missing, limiting evaluation of associations between the CHAI biomarker and these variables. Limited granular systemic treatment data were available to explore the implications of the CHAI biomarker for treatment decision-making. To address some of these limitations, further study of the biomarker is warranted to establish its validity and clinical utility. In specific, future efforts will seek to assess the performance of the biomarker in a cohort beyond the TCGA that includes patients receiving the full range of standard of care treatment regimens, to confirm its relevance in the setting of contemporary CRC management. Evaluation of the biomarker in multicenter CRC cohorts receiving adjuvant chemotherapy will ground and clarify the utility of this prognostic biomarker in the stage II and III contexts where risk stratification is foundational.

## Supplementary Material

Suppl 1 PFI by lymphovascular invasion.

## Data Availability

All histologic data from the TCGA used for analysis are publicly available from the TCGA. Biomarker calls may be made available to qualified researchers upon reasonable request to the corresponding author.
