# Supplementary material for: Development of a Computational Histology Artificial Intelligence-Powered Prognostic Biomarker in Colorectal Cancer in The Cancer Genome Atlas
Source: World J Oncol. 2026 Sep 4;17(5):614–22. doi: 10.14740/wjon2823 (PMC13568777; doi:10.14740/wjon2823)
Supplement: Supplementary file 1 [file wjon-17-05-614-s001.docx]

**Suppl 1.** PFI by lymphovascular invasion. Kaplan– Meier estimates of PFI are stratified by lymphovascular invasion status (LVI) in (a) all validation cohort patients (n = 382; this includes all patients with LVI status reported), (b) stage II patients (n = 139), and (c) stage III patients (n = 116). P values correspond to log-rank tests. AJCC: American Joint Committee on Cancer; CI: confidence interval; PFI: progression-free interval; LVI: lymphovascular invasion.
